# Supplementary figures and images for: Environmental determinants of E. coli, link with the diarrheal diseases, and indication of vulnerability criteria in tropical West Africa (Kapore, Burkina Faso)
Source: PLoS Negl Trop Dis. 2021 Aug 17;15(8):e0009634. doi: 10.1371/journal.pntd.0009634 (PMC8370611; doi:10.1371/journal.pntd.0009634)

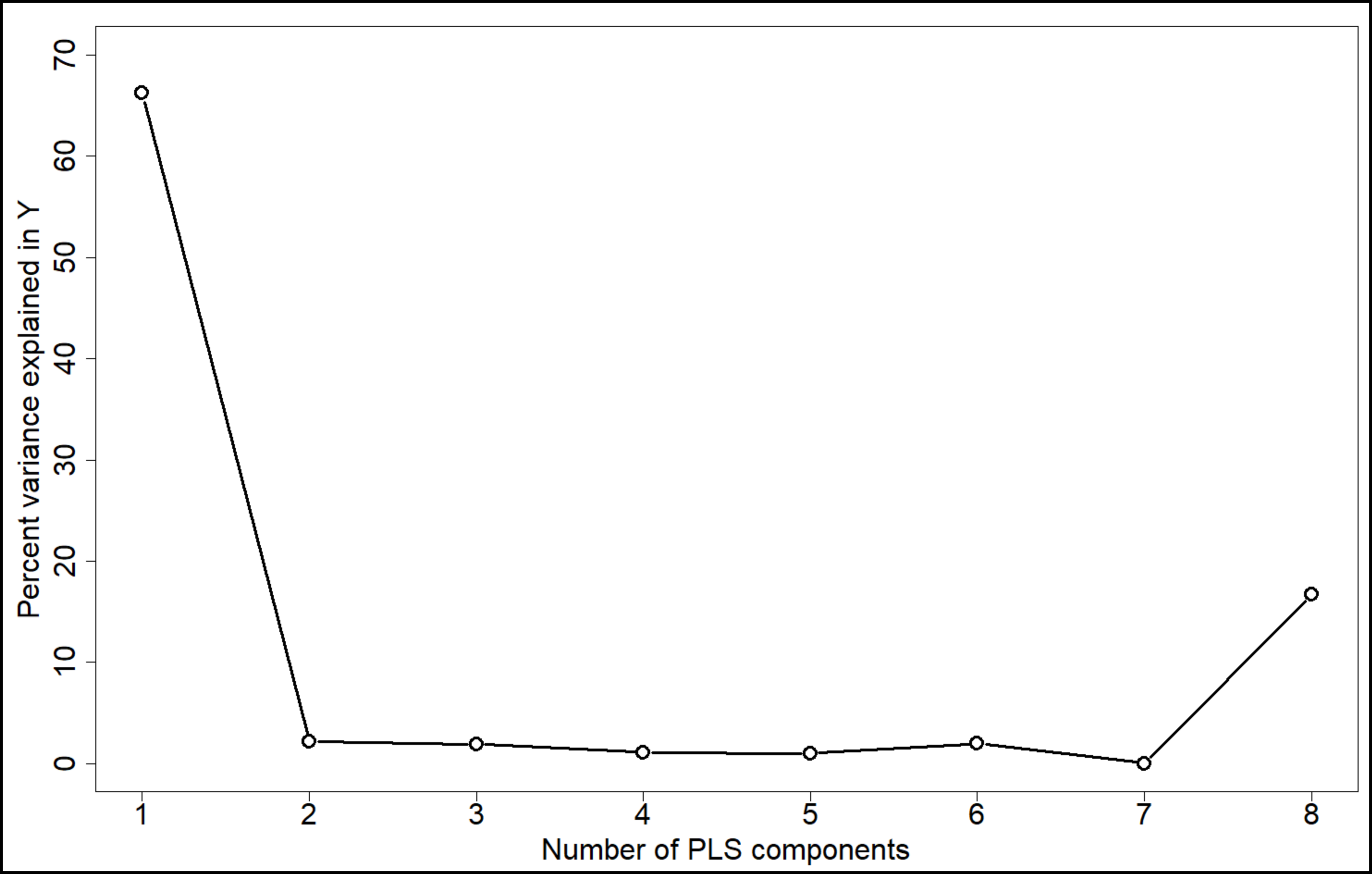

Supplement: S1 Fig — (TIF) [file pntd.0009634.s001.tif]

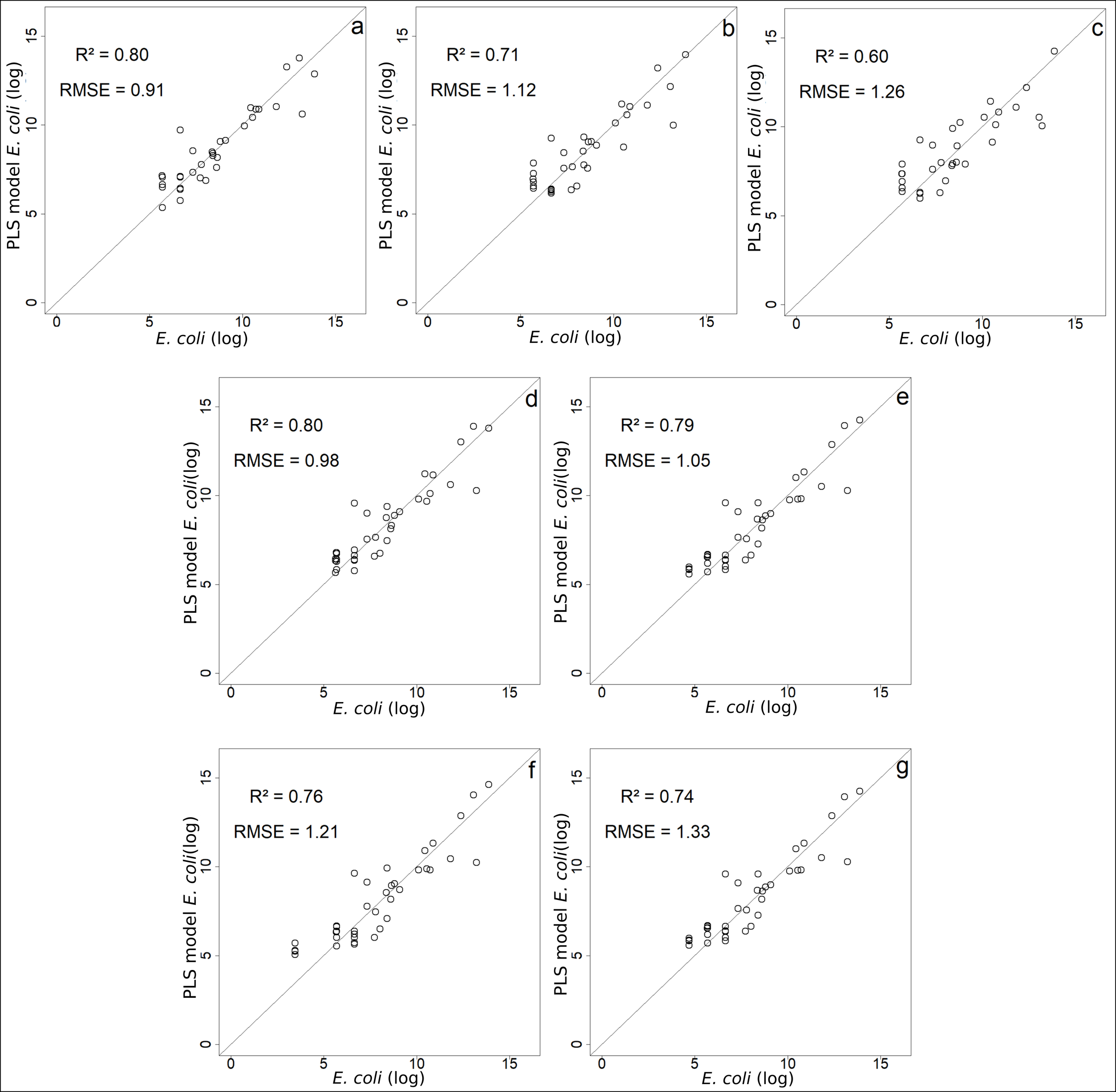

Supplement: S2 Fig — Comparison of models’ predictions with observed E. coli (log values) (a) PLS model with all environmental variables without individuals 31–34; (b) PLS model with NIR band, SPM and Weekly rainfall without individuals 31–34; (c) PLS model with satellite variables only (NIR band and Weekly rainfall) without individuals 31–34; (d) PLS model with all environmental variables and value 49 assigned for the values less than 50 MPN 100mL-1; (e) PLS model with all environmental variables and value 25 assigned for the values less than 50 MPN 100mL-1; (f) PLS model with all environmental variables and value 10 assigned for the values less than 50 MPN 100mL-1; (g) PLS model with all environmental variables and value 5 assigned for the values less than 50 MPN 100mL-1. (TIF) [file pntd.0009634.s002.tif]

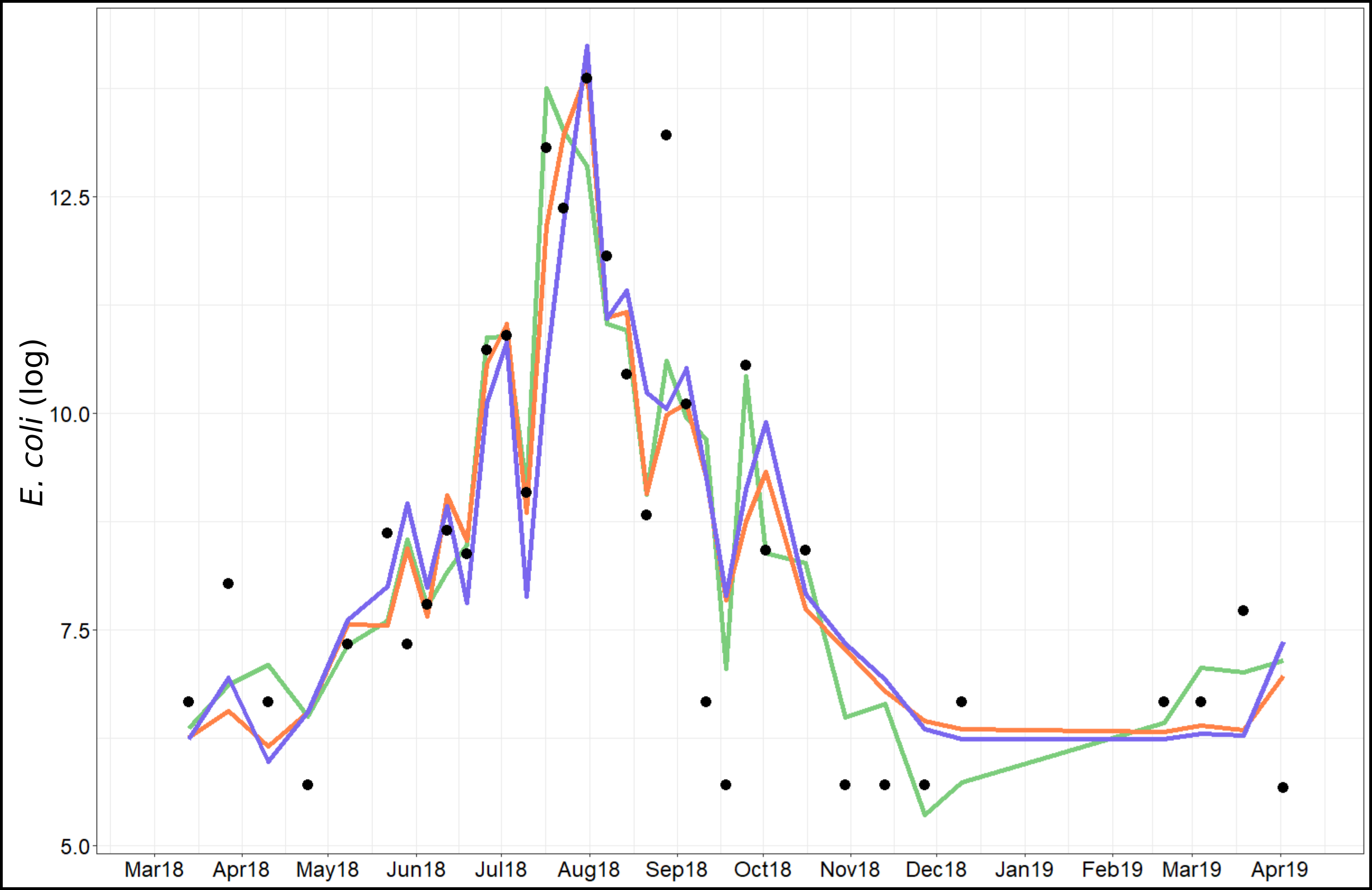

Supplement: S3 Fig — Observed E. coli (black point) and predicted E. coli (a) without individuals 31–34 (black line for all variables, red line for NIR, SPM and Rainfall variables, blue line for satellite variables). (TIF) [file pntd.0009634.s003.tif]

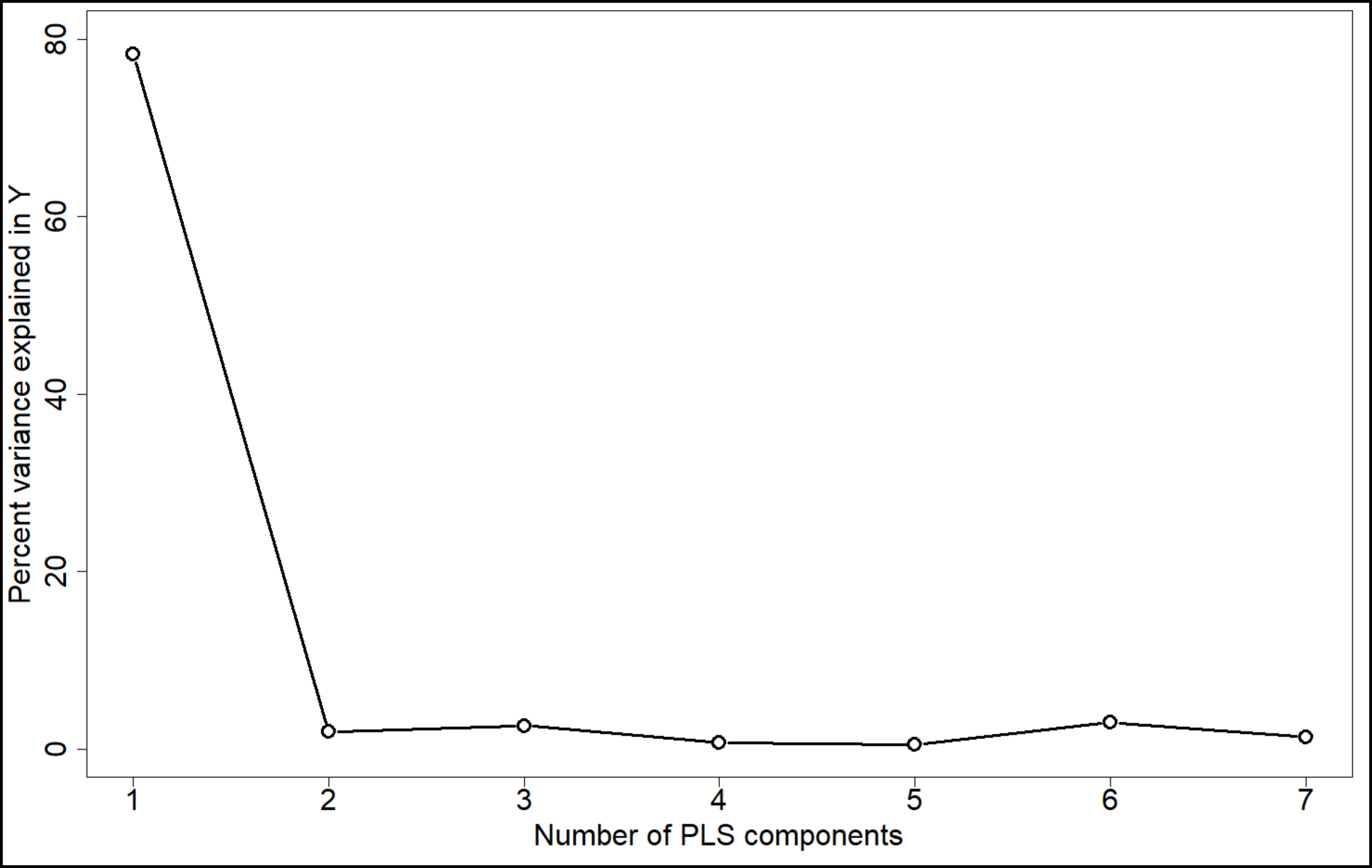

Supplement: S4 Fig — (TIF) [file pntd.0009634.s004.tif]

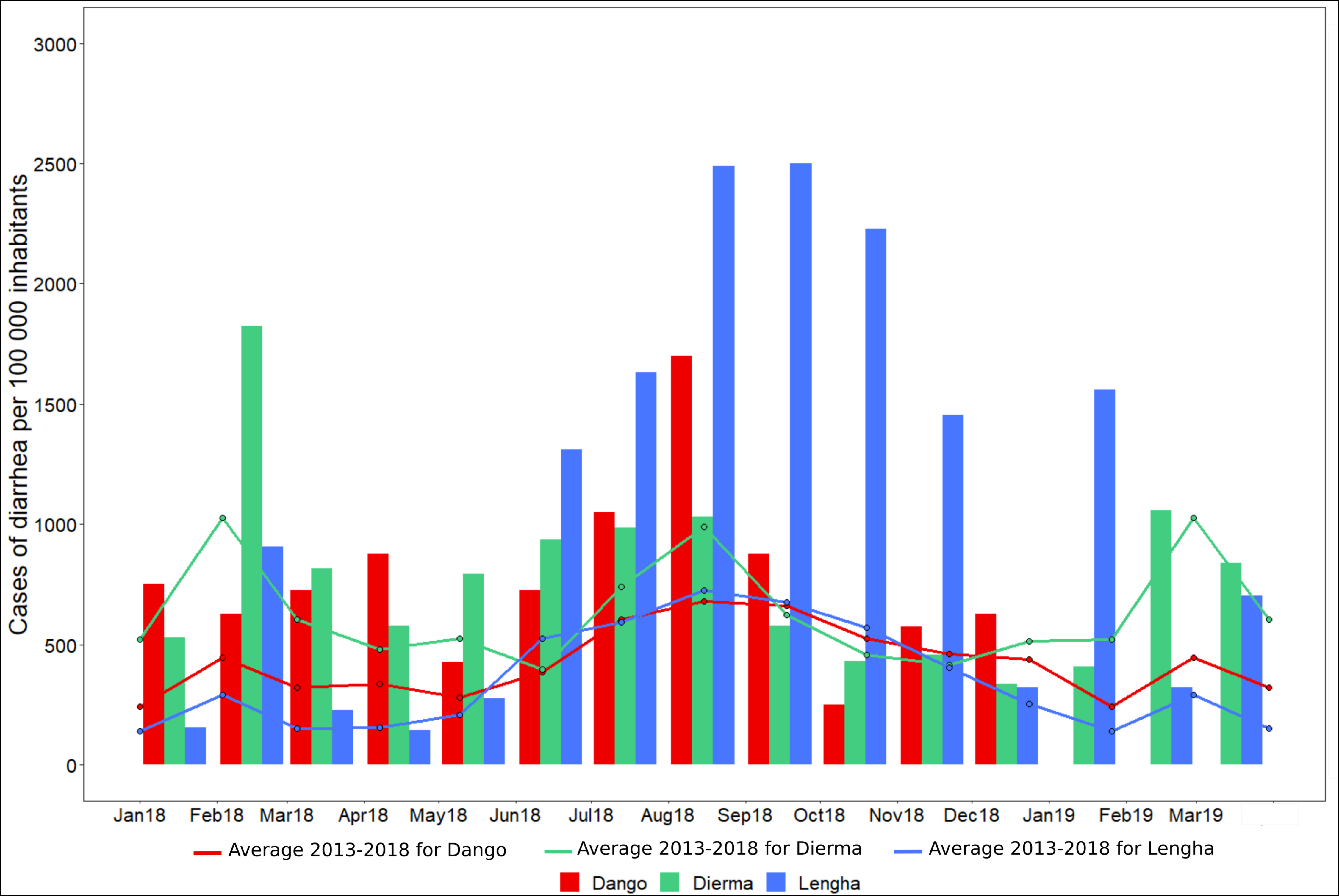

Supplement: S5 Fig — (TIF) [file pntd.0009634.s005.tif]
