# Supplementary material for: Environmental determinants of E. coli, link with the diarrheal diseases, and indication of vulnerability criteria in tropical West Africa (Kapore, Burkina Faso)
Source: PLoS Negl Trop Dis. 2021 Aug 17;15(8):e0009634. doi: 10.1371/journal.pntd.0009634 (PMC8370611; doi:10.1371/journal.pntd.0009634)
Supplement: S1 Table — (PDF) [file pntd.0009634.s006.pdf]

Variable	Percent
**SPM**	**79**
**Weekly Rainfall**	**71**
**Water level**	**4**
**Cumulative rainfall**	**9**
**Nb pixel water**	**3**
**NDVI**	**32**
**NIR band**	**83**
**Daily rainfall**	**37**

S1 Table. Percentage of the variability of *E. coli* explained by each of the variables for the 1st component
