# Supplementary material for: Environmental determinants of E. coli, link with the diarrheal diseases, and indication of vulnerability criteria in tropical West Africa (Kapore, Burkina Faso)
Source: PLoS Negl Trop Dis. 2021 Aug 17;15(8):e0009634. doi: 10.1371/journal.pntd.0009634 (PMC8370611; doi:10.1371/journal.pntd.0009634)
Supplement: S2 Table — (PDF) [file pntd.0009634.s007.pdf]

Variable	Percent
**SPM**	**68**
**Rainfall**	**80**
**Water level**	**4**
E. coli	**73**
**Nb pixel water**	**0.6**
**NDVI**	**67**
**NIR**	**83**

S2 Table. Percentage of the variability of cases of diarrhea explained by each of the variables for the 1st component
